# The change in dental fear and anxiety toward dental examinations among preschool children in Hong Kong. 18-months longitudinal study

**DOI:** 10.3389/froh.2026.1806157

**Published:** 2026-06-01

**Authors:** Phoebe Pui Ying Lam, Sarrah S. F. S. Almarzouq, Cynthia Yiu

**Affiliations:** Paediatric Dentistry, The University of Hong Kong, Pokfulam, Hong Kong, SAR China

**Keywords:** child, dental anxiety, dental care, dental fear and anxiety, preschool children

## Abstract

**Purpose:**

Dental fear and anxiety (DFA) can profoundly impact on children's oral health and pose challenges for dental care providers due to behavioural issues. This 18-month cohort study aimed to investigate the changes in the prevalence and levels of DFA among preschool children towards dental examination in a familiar kindergarten setting and different potential risk factors that may influence the changes in dental fear levels among preschool children.

**Methods:**

Parental questionnaires consisting the child's demographic information, oral health status, socio-economic background, previous dental experiences, parental and child self-reported DFA were administered. During each biannual review, the children underwent examinations for plaque scores and dental caries. Their DFA was evaluated based on behaviours and perceived pain levels both before and immediately after the clinical examination using Frankl Scale (FBRS) and Venham Scale (VBRS). The children's perceived pain levels were self-assessed using Wong-Baker Faces Pain Scale (WBFS).

**Results:**

A total of 300 healthy kindergarten children aged 2–5 years were enrolled, with 209 children being reviewed at 18 months. A notable increase in children's cooperativeness pre-operatively and post-operatively over time was observed when assessed using FBRS (*p* < 0.001) and VBRS (*p* < 0.05), but not for self-rated WBFS. Following multiple imputation, children with higher dft and dt scores were significantly associated with pre-operative uncooperative behaviours. Other sociodemographic factors, clinical findings, or reported dental fear and anxiety (DFA) by parents and children were not significantly linked to uncooperative behaviours either pre-operatively or post-operatively.

**Conclusion:**

The prevalence of DFA among preschool children towards dental examination in familiar kindergarten settings decreased significantly over time, suggesting the positive impact of repeated, non-threatening dental encounters. Past caries experience and untreated dental decay remained as significant factors associated with DFA.

## 1 Introduction

Dental fear is the reaction elicited by a particular threatening stimulus in a dental environment, while dental anxiety is an amplified sense of apprehension linked to the fear of potential discomfort associated with dental visits. Dental fear and anxiety (DFA) are common issues impacting individuals of all ages worldwide (1). Studies have reported the prevalence of dental fear and anxiety among adults to be 10.2–21.2% (2), whereas among children, it varies from 4% to 98% (3).

DFA in children can have significant and enduring effects on their oral health (4). Children experiencing DFA may face obstacles in accessing essential dental care due to behavioural challenges and avoidance of dental visits, leading to increased difficulties, stress, and time required for dental healthcare providers to deliver necessary treatments (1). Research indicates that children with DFA are more likely to exhibit poorer oral hygiene, higher rates of dental decay, oral infections, and reduced oral health-related quality of life compared to their peers. As these effects may persist into adulthood, it is crucial to identify the potential factors that influence DFA among young children.

DFA is a multifaceted issue influenced by various sociodemographic and clinical factors, including the age of the patient and parental fear (5, 6). Age is recognised as a significant factor in DFA, with younger children possessing lower cognitive abilities to comprehend the unfamiliar aspects of dental visits (6). However, the majority of studies on risk factors have been cross-sectional, making it challenging to assess potential changes in DFA among younger children, particularly those who initially exhibit DFA. Another important factor to consider is parental DFA, which has been shown to potentially impact children's fear levels. While conflicting findings exist regarding the relationship between parental DFA and DFA among school children and adolescents (7, 8), longitudinal research on the influence of parental dental fear and other associated factors on younger preschool children is currently scarce (5, 6).

This cohort study aimed to investigate the changes in the prevalence and levels of dental fear among preschool children towards dental examination in a familiar kindergarten and nursery setting over an 18-month period. Additionally, the study aimed to identify different risk factors that may influence the changes in dental fear levels among preschool children.

## 2 Material and methods

### 2.1 Study design and settings

The 18-month cohort study was conducted in 11 kindergartens of Hong Kong, China and reported in accordance with the Strengthening the Reporting of Observational Studies in Epidemiology (STROBE) guidelines. Preschool children were recruited at kindergarten grade 1–2 and reviewed biannually for 18 months at their respective kindergartens from September 2022 to August 2024. Ethical approval was sought from the Institutional Review Board of the University of Hong Kong/Hospital Authority Hong Kong West Cluster (UW23-039).

### 2.2 Participants

Healthy kindergarten children aged 2–5 years were recruited in this study. Invitation letters were sent to their respective kindergartens and distributed to their parents, explaining the purposes and objectives of the study. Only those children whose parents had signed the informed consent were included in this study. Children aged over 71 months at baseline, with known medical or psychological history, or motor impairment were excluded.

### 2.3 Variables and data measurement

The level of DFA among preschool children before and immediately after the dental examination at the kindergarten were evaluated by researcher (S.A) with Frankl behaviour rating scale (FBRS) (9) and Venham behavioural rating scale (VBRS) (10). The pain perceived by children was also assessed with a self-rated Wong-Baker Faces Scale (11). The former two operator-rated scales were chosen as they were well-validated, simple to administer and with clearly defined criteria (9). The use of the WBFS was chosen due to its extensive history of use, strong supporting evidence, and validation (11).

A self-reported questionnaire was administered to parents, which included the Corah Dental Anxiety Scale (CDAS) (12, 13) and Children's Fear Survey Schedule—Dental Subscale (CFSS-DS) (14) to assess both the children's and parents' DFA. The CDAS comprises four questions that describe the parent's DFA in response to various dental scenarios. The maximum score of CDAS is 20, where a lower score indicates lower Dental Fear and Anxiety (DFA). Parents scoring between 9 and 12, 13–14, and 15 or above were categorised as experiencing moderate, high, and severe anxiety, respectively (12, 13). On the other hand, the CFSS-DS consists of 15 questions with 5-point Likert scales represented by faces, ranging from “not afraid at all” (1) to “very much afraid” (5, 14). Parents were requested to assist their children in completing the questionnaire. Parents and children who self-reported their DFA via the CDAS and CFSS-DS questionnaires were eligible to receive a toothbrushing souvenir pack as a token of appreciation. Sociodemographic data and information on the child's oral health habits were collected via a validated questionnaire (15).

Children were brought to the examination room by their teachers and were asked by a research assistant to rate their perceived state of pain levels using WBFS (11) before the start of the oral examinations. The children were asked to lie supine on kindergarten tables covered with blankets and plastic table cloth. An oral examination was subsequently performed by a single dental examiner (first author), who wore full personal protective equipment and utilised disposable mirrors attached to intraoral light-emitting diode (MirrorLite, Kudos Crown Ltd, Hong Kong) along with a cotton swab. The examiner, a paediatric dentist, employed various non-pharmacological behavioural management techniques during the examination. These techniques included tell-show-do, verbal praises for positive reinforcement during the examination, distraction through cartoon playing with an electronic device fixed with a portal hanger, and tangible stickers as positive reinforcement upon completion of the procedures by the children.

Clinical factors including oral hygiene and dental caries experience were also measured. During the dental examination, Visual Plaque Index (VPI) (16) was initially documented. Subsequently, following cleaning and drying using gauze and cotton swabs, the number of decayed, missing and filled teeth (dmft) and surfaces (dmfs) due to dental caries (17), and the ICDAS II score (18) of each tooth surface were recorded. The examiner underwent training and calibration by an epidemiologist, resulting in Kappa ratings of 0.774 for VPI, 0.964 for dmft, and 0.834 for ICDAS II score in terms of intra-examiner reliability (19).

Another researcher (second author) assessed the children's DFA and cooperativeness during the baseline examination with and VBRS. A research assistant then requested the child to provide their WBFS rating again after the dental examination as they were leaving the examination room.

### 2.4 Exposure and potential confounders

At baseline, parents were also asked to complete a questionnaire to investigate different potential factors associated with DFA. This included children's personal information including gender and medical conditions, their oral hygiene and dietary practices, as well as their family socio-economic background. The levels of dental fear and anxiety in both parents and children were assessed using the self-reported Corah Dental Anxiety Scale (12).

### 2.5 Sample size calculation

The prevalence of dental anxiety reported by Yon et al. (6) among preschool children measured with Frankl scale was 6% (6). The targeted precision and confidence interval set as 5% and 95% respectively, and the dropout rate assumed to be 10%, a minimal of 264 students are required at baseline.

### 2.6 Follow up evaluations

Participants were reviewed biannually in the same kindergarten settings by the same researchers. Pre- and post-operative DFA, as well as dental examination for VPI, dmft and ICDAS scores were measured similarly as baseline.

### 2.7 Outcome measures

The primary outcome of study was the change in the prevalence and level of dental fear level of preschool children towards dental examination over time (6th, 12th, and 18th months), measured with VBRS. The secondary outcome was the measurement of children's cooperativeness and anxiety with FBRS and pain level with WBFS.

### 2.8 Data processing and analysis

Data analyses were conducted using SPSS® Statistics version 28.0 (SPSS Inc, Chicago, IL, USA). Children with missing data were excluded from the analysis. Chi-square tests and bivariate analyses were used to assess the difference of prevalence of dental fear measured with FBRS, VBRS and WBFS at baseline, 6 months, 12 months and 18 months. The effects of demographic, oral health and clinical factors on children DFA were analysed with a logistic regression analysis, or bivariate analyses depending on the proportion of events.

To handle the missing data at 18 months, multiple imputation by chained equations (MICE) was carried out before bivariate analyses and logistic regression for DFA at 18 months. This method utilised a sequential approach where each variable with missing values was modelled conditionally on all other available observed variables. Imputation model included socio-demographic background, oral health habits, baseline clinical findings, parental and child DFA. In missing imputation, logistic regression was employed for binary variables while predictive mean matching (PMM) with the closest 5 predictions was employed for continuous variables to preserve their distribution properties and handle non-normality. Totally twenty imputed datasets were generated with maximum 10 iterations. Analyses were conducted on each imputed data sets. For parametric analyses, results were pooled following Rubin's rules (20). For Chi-square tests, results were combined performed using SAS OnDemand for academics (SAS Institute Inc., Cary, NC, USA) with the %COMBCHI macro (21) following Schafer's method for combining chi-square statistics (22). For non-parametric analyses due to the lack of established pooling methods of Mann–Whitney *U*-tests, results are presented as the proportion of significant findings across the 20 imputations, along with the range of *p*-values obtained. The level of statistical significance for all tests was set at 5%.

## 3 Result

**Figure 1:**
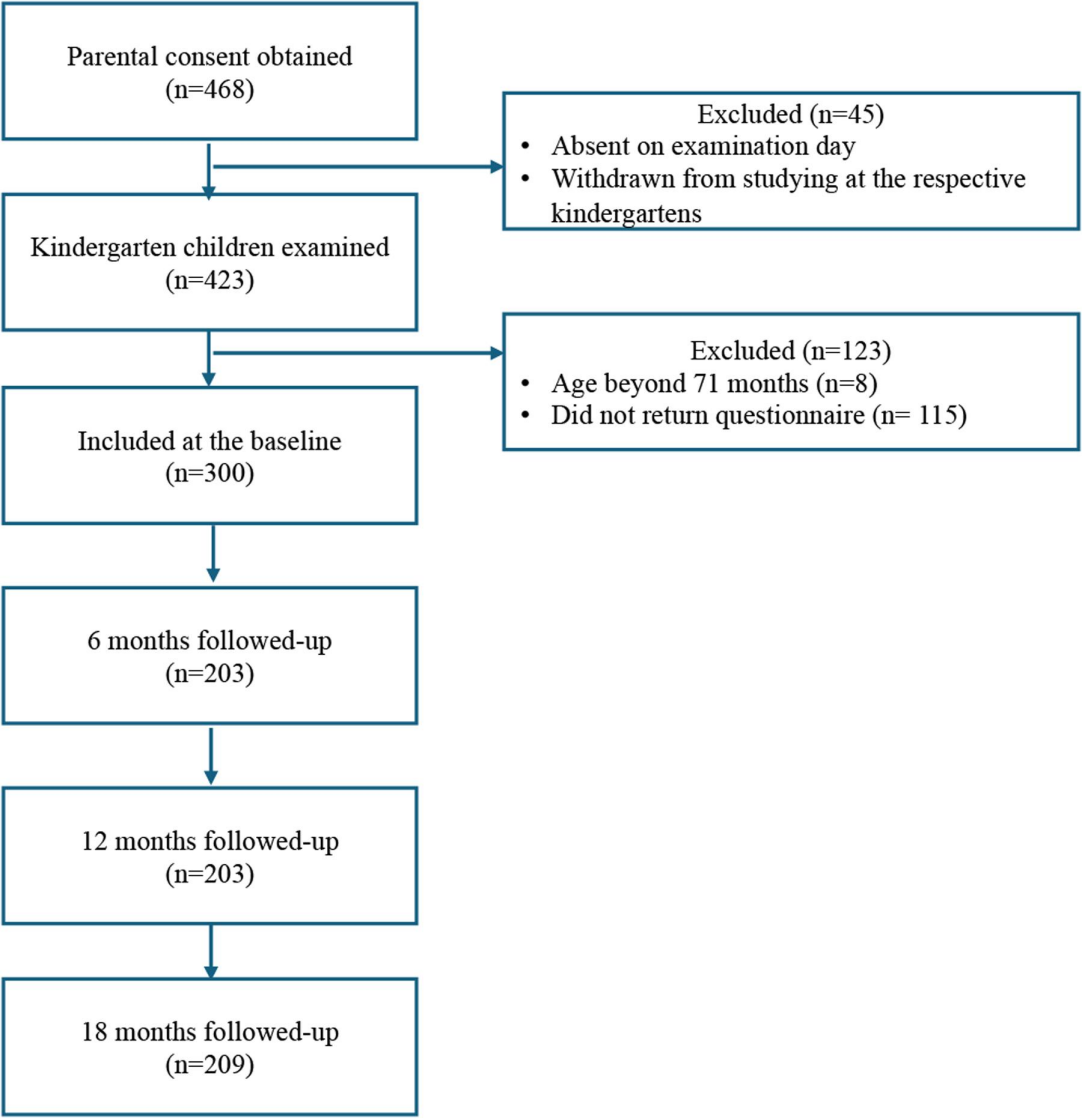
Flow of subjects.

Parental consent was obtained from 468 children attending kindergarten grade 1 from 11 kindergartens. Only 423 students were examined at baseline as 45 students were either absent or withdrawn from studying in the respective kindergartens. One-hundred-and-fifteen students were further excluded from the analyses as they did not complete the questionnaires, and eight students were excluded from the study as they were over 6 years old at the start of the study. Hence only 300 students were included at baseline. At 6 months and 12 months, 203 children were reviewed, whereas the remaining 97 students were either absent or left their kindergartens. At 18 months, 209 children remained and assessed. Figure 1 details the flow of the sample population.

The demographic profile, oral health status and cooperation levels of the study participants are presented in Table 1. Approximately half of the children (54.0%, *n* = 162) were boys, and the majority (94.7%, *n* = 284) were born in Hong Kong. Most children (61.0%, *n* = 183) brushed their teeth twice a day, with around 46.3% (*n* = 139) using fluoridated toothpaste and more than half of them (58.7%, *n* = 176) always being supervised during toothbrushing. Almost half of the children (47%, *n* = 141) snacked twice or more per day, meanwhile the majority (67.0%, *n* = 201) never had a habit of using a bottle at night. Additionally, most children (89.0%, *n* = 267) had never visited a dentist. Most of the parents belonged to the 30–39 years old age group (father: 49.3%, *n* = 148; mother: 65.3%; *n* = 196). More than one third of the children (32.3%, *n* = 97) had a family monthly income ranging from HKD 20,000 to 30,000. Over 40% of the parents had tertiary or post-secondary education level (fathers 46.0%, *n* = 138; mothers 42.0%, *n* = 126). Almost half of the children had at least 1 sibling (58.3%, *n* = 175) while around one-third of the children were the only children (31.0%, *n* = 93). The mean age of the children was 49.6 months ± 6.2 months.

**Table 1:** Baseline demographic profile, oral health status and cooperativeness of study participants (*n* = 300).

Factors	Loss to follow-up (*N* = 91)	Reviewed at 18 months (*N* = 209)	
Child demographic profile	% (*n*)	% (*n*)	*p*-value
Child gender			0.471^a^
Male	57.1 (52)	52.6 (110)	
Female	42.9 (39)	47.4 (99)	
Place of birth			0.788^b^
Hong Kong	95.6 (87)	94.7 (198)	
Mainland or others	4.4 (4)	3.8 (8)	
Undisclosed	0.0 (0)	1.4 (3)	
Father age range			0.381^b^
20–29 years	7.7 (7)	3.3 (7)	
30–39 years	45.1 (41)	51.2 (107)	
40–49 years	36.3 (33)	33.5 (70)	
50 or above	7.7 (7)	10.0 (21)	
Undisclosed	3.3 (3)	1.9 (4)	
**Mother age range**			0.101^b^
20–29 years	19.8 (18)	9.6 (20)	
30–39 years	60.4 (55)	67.5 (141)	
40–49 years	17.6 (16)	21.1 (44)	
50 or above	0.0 (0)	0.0 (0)	
Undisclosed	2.2 (2)	1.9 (4)	
**Family income (HKD per month)**			0.240^a^
<$20.000	24.2 (22)	16.7 (35)	
$20,001–$30,000	36.3 (33)	30.6 (64)	
$30,001–$40,000	9.9 (9)	15.3 (32)	
$40,000 or above	20.9 (19)	29.2 (61)	
Undisclosed	8.8 (8)	8.1 (17)	
**Father education level**			0.381^a^
Primary school or less	1.1 (1)	2.4 (5)	
Secondary school	57.1 (52)	47.4 (99)	
Post-secondary/University	35.2 (32)	45.0 (94)	
Post-graduate or above	4.4 (4)	3.8 (8)	
Undisclosed	2.2 (2)	1.4 (3)	
**Mother's education level**			0.663^a^
Primary school or less	5.5 (5)	2.9 (6)	
Secondary school	49.5 (45)	51.7 (108)	
Post-secondary/University or above	35.2 (32)	37.8 (79)	
Post-graduate or above	5.5 (5)	4.8 (10)	
Undisclosed	4.4 (4)	2.9 (6)	
**Number of siblings**			0.068^a^
Only child	34.1 (31)	29.7 (62)	
1 sibling	35.2 (32)	49.3 (103)	
2 siblings	14.3 (13)	9.1 (19)	
3 or more siblings	1.1 (1)	3.3 (7)	
Undisclosed	15.4 (14)	8.6 (18)	
	**Mean (SD)**	**Mean SD**	***p*-value**
**Age (years old)**	46.8 (6.2)	50.8 (5.9)	<0.001^c^^,^***
***Oral habits***	**% (n)**	**% (n)**	***p*-value**
**Brushing frequency**			0.002^b^^,^**
Less than once a day	5.5 (5)	7.2 (15)	
Once a day	46.2 (42)	24.9 (52)	
Twice a day	47.3 (43)	67.0 (140)	
More than twice a day	1.1 (1)	1.0 (2)	
**Supervised toothbrushing**			0.019^b^^,^*
Never	3.3 (3)	7.7 (16)	
Once a day	26.4 (24)	38.3 (80)	
Twice or more a day	69.2 (63)	54.1 (113)	
Undisclosed	1.1 (1)	0.0 (0)	
**Toothpaste**			
No toothpaste	9.9 (9)	6.2 (13)	0.023^a^^,^*
Child non-fluoridated toothpaste	48.4 (44)	33.0 (69)	
Child fluoridated toothpaste	31.9 (29)	51.7 (108)	
Adult fluoridated toothpaste	1.1 (1)	0.5 (1)	
Uncertain or undisclosed	8.8 (8)	8.6 (18)	
**Snacking frequency**			
Less than once a day	14.3 (13)	16.7 (35)	0.287^a^
Once a day	44.0 (40)	34.0 (71)	
Twice a day	34.1 (31)	35.9 (75)	
Over twice	7.7 (7)	13.4 (28)	
**Night bottle habit**			
Never	60.4 (55)	69.9 (146)	0.210^a^
Yes	15.4 (14)	9.6 (20)	
Previously yes but winded up recently	24.2 (22)	20.6 (43)	
**Previous dental visit**			
No	86.8 (79)	90.0 (188)	0.548^a^
Yes	13.2 (12)	10.0 (21)	
**Oral health parameters**			
	**Mean (SD)**	**Mean (SD)**	***p*-value**
VPI score (%) (*N* = 295)	35.2 (16.9)	35.2 (17.1)	0.970^c^
dft score	1.4 (3.4)	1.4 (2.9)	0.242^d^
dt score	1.4 (3.4)	1.4 (2.8)	0.247^d^
ft score	0.0 (0.0)	0.0 (0.1)	0.251^d^
**Baseline anxiety and pain level**			
	**% (n)**	**% (n)**	***p-value***
***Pre-intervention***			
**Frankl scale (FBRS)**			
Uncooperative (1–2)	25.3 (23)	11.5 (24)	0.003**
Cooperative (3–4)	74.7 (68)	88.5 (185)	
**Venham scale (VBRS)**			0.006**
Cooperative (0)	73.6 (67)	87.6 (183)	
Uncooperative (≥ 1)	26.4 (24)	12.4 (26)	
**Wong-Baker scale (WBFS)**			<0.001***
No hurt (0)	68.1 (62)	85.2 (178)	
Hurts (2–10)	31.9 (29)	14.8 (31)	
***Post-intervention***			
**Frankl scale (FBRS)**			0.004**
Uncooperative (1–2)	17.6 (16)	5.7 (12)	
Cooperative (3–4)	82.4 (75)	94.3 (197)	
**Venham scale (VBRS)**			0.021*
Cooperative (0)	80.2 (73)	90.9 (190)	
Uncooperative (≥1)	19.8 (18)	9.1 (19)	
**Wong-Baker scale (WBFS)**			<0.001***
No hurt (0)	71.4 (65)	88.5 (185)	
Hurts (2–10) or do not answer	28.6 (26)	11.5 (24)	
**Self-reported DFA**			
**Parental reported DFA**	*N* = 45	*N* = 121	
	**Mean (SD)**	**Mean (SD)**	***p-value***
Parental CDAS	9.2 (2.7)	9.0 (2.7)	0.799^c^
	**% (n)**	**% (n)**	***p-value***
Low fear (<9)	40.0 (18)	51.2 (62)	0.312^a^
Medium fear (9–12)	46.7 (21)	33.9 (41)	
High to severe fear (13–20)	13.3 (6)	14.9 (18)	
			
**Child reported DFA**	*N* = 42	*N* = 119	
	**Mean (SD)**	**Mean (SD)**	***p-value***
Child CFSS-DS	40.1 (12.0)	42.3 (11.3)	0.310^c^

a *p*-values derived from Chi-square statistics.

b *p*-values derived from Fisher-exact statistics.

c *p*-value derived from *t*-test for independent samples.

d *p*-value derived from Mann–Whitney *U*-tests.

†† include the findings of “cannot tell”.

* *p* < 0.05.

** *p* < 0.01.

*** *p* < 0.001.

From the clinical oral examination, the prevalence of children with dental caries was 31%, 35% and 41% at baseline, 6 months and 12 months. The dft scores of children were 1.4 ± 3.0 at baseline, 1.6 ± 3.1 at 6 months, 2.1 ± 3.6 at 12 months, and 2.3 ± 3.5 at 18 months. While the mean VPI scores of children were 35.2 ± 17.0 at baseline, 31.3 ± 16.4 at 6 months, 36.2 ± 15.7 at 12 months, and 38.3 ± 15.3 at 18 months.

The dropout rate at the 18-month review was 30.3%, with 91 out of 300 subjects being lost to follow up (Table 1). A significant difference was observed in sociodemographic factors between those who were lost to follow up and those who remained in the study. It was found that a significantly higher proportion of children who were reviewed at 18 months brushed their teeth twice per day (*p* = 0.002), did not receive or only occasionally received supervised toothbrushing (*p* = 0.019), use fluoridated toothpaste (*p* = 0.023), and were significantly younger (*p* < 0.001). Additionally, significantly higher proportions of children who were reviewed at 18 months demonstrated total cooperation when measured with FBRS (pre-operative: *p* = 0.003; post-operative: *p* = 0.004) and VBRS (pre-operative: *p* = 0.006; post-operative: 0.021).

Only 170 DFA questionnaires were received, out of which 166 complete parental CDAS responses were included for analysis. The mean score for parental CDAS was 9.0 (±SD 2.7), with 48.2% (*n* = 80) of parents reporting low DFA, 37.3% (*n* = 62) reporting moderate DFA, and 14.5% (*n* = 24) reporting high to severe DFA. In terms of DFA among children, the CFSS-DS items “2.2 doctor” (1.8 ± SD 1.0) and “2.5 having to open your mouth” (1.9 ± SD 1.2) were associated with the least anxiety, while “2.8 the dentist drilling” (3.8 ± SD 1.4) and “2.9 the sight of dentist drilling” (3.5 ± SD 1.3) were linked to the most distress. The mean CFSS-DS score was 41.7 (±SD 11.5) among 156 children who have responded to all 15 items. No difference in CDAS (*p* = 0.799) and CFSS-DS (*p* = 0.310) were found between the participants reviewed at 18 months and those lost to follow up (Table 1).

Table 2 shows the changes in children's pre-operative and post-operative cooperativeness and self-reported pain level over time. A significant increase in children's pre-operative cooperativeness over time were found when evaluated with FBRS (*p* < 0.001) and VBRS (*p* < 0.001). When measured with FBRS, a significantly higher proportion of children displayed more cooperation at 6 months (96.1%), 12 months (98.0%) and 18 months (99.5%) compared to the baseline (84.3%), and the proportion of children being cooperative at 18 months were significantly higher than 6 months, but no difference was found in the cooperativeness of children between 6 and 12 months, as well as between 12 and 18 months. When measured with VBRS, children at 18 months (98.1%) were significantly more cooperative that at baseline (83.3%) and 6 months (95.6%), but no significant difference was found between the pre-operative cooperativeness of children at 6 months (95.6%) and 12 months (94.1%), and between 12 months (94.1%) and 18 months (98.1%) (Table 2).

**Table 2:** Change in cooperativeness over time.

Assessment scale	Baseline (1) (n = 300) % (n)	6 months (2) (n = 203) % (n)	12 months (3) (n = 203) % (n)	18 months (4) (n = 209) % (n)	Complete case	P-value	Pairwise Comparisons
*Pre-operative dental anxiety and pain*							
**Frankl scale**	***+ve (3–4)***	***+ve (3–4)***	***+ve (3–4)***	***+ve (3–4)***			
	84.3 (253)a	96.1 (195)b	98.0 (199)bc	99.5 (208)c	***n*** ***=*** ***118***	***<0***.***001***^a^^,^***	***(2)*** ***>*** ***(1)***
							***(3)*** ***>*** ***(1)***
							***(4)*** ***>*** ***(1)***
**Venham scale** **Am**	***+ve (0)***	***+ve (0)***	***+ve (0)***	***+ve (0)***	***n*** ***=*** ***118***	***<0***.***001***^a^^,^***	***(4)*** ***>*** ***(1)***
	83.3 (250)a	95.6 (194)a	94.1 (191)ab	98.1 (205)b			***(3)*** ***>*** ***(1)***
**Wong Baker scale°**	***+ve (0)***	***+ve (0)***	***+ve (0)***	***+ve (0)***	***n*** ***=*** ***118***	***0***.***757***^a^	
	80.0 (240)	85.2 (173)	89.2 (181)	85.2 (178)			
*Post-operative dental anxiety and pain*							
**Frankl scale**	***+ve (3–4)***	***+ve (3–4)***	***+ve (3–4)***	***+ve (3–4)***	***n*** ***=*** ***118***	***<0***.***001***^a^^,^***	***(4)*** ***>*** ***(1)***
	90.7 (272)a	96.1 (196)a	99.5 (202)b	99.5 (208)b			***(3)*** ***>*** ***(1)***
**Venham scale**	***+ve (0)***	***+ve (0)***	***+ve (0)***	***+ve (0)***			
	87.7 (263)a	95.6 (194)a	98.5 (200)b	96.7 (202)b	***n*** ***=*** ***118***	***0***.***011***^a^^,^**	***(3)*** ***>*** ***(1)***
**Wong Baker scale°**	***+ve (0)***	***+ve (0)***	***+ve (0)***	***+ve (0)***	***n*** ***=*** ***118***	***0***.***141***^a^	
	83.3 (250)	87.7 (178)	94.6 (192)	90.4 (189)			

a *p*-value derived from Cochran's *Q*-Test.

* *p* *<* *0.05.*

** *p* *<* *0.01.*

* *p* *<* *0.001.*

+ve = positive behaviours.

Similar trend was observed when comparing children's post-operative cooperativeness over time. The proportions of cooperative children were significantly higher at 12 and 18 months compared to baseline and 6 months when assessed with both FBRS (<0.001) and VBRS (<0.05), but no significant difference was observed between baseline and 6 months, as well as between 12 months and 18 months (Table 2).

For anticipated pain among children, there was no significant difference in pre-operative WBFS (*p* = 0.757) and post-operative WBFS (*p* = 0.141) reported by children over time (Table 2). The proportion of children reported “no hurt” pre-operatively and post-operatively were similar at baseline, 6 months, 12 months and 18 months. Multivariate analyses with logistic regression was not used as the prevalence of dental fear represented by uncooperative behaviours with VBRS was less than one-fifth. Bivariate analysis of each potential risk factor was used instead.

At baseline, children who only brushed once per day were more likely to be pre-cooperative before and immediately after the dental exam than those who brushed twice or less than once per day. Post-operatively, boys were more likely to be uncooperative than girls (*p* = 0.034), as well as children whose mother only receive primary education (*p* = 0.044). Other clinical and sociodemographic factors was found to have no significant association with cooperativeness of the children (*p* > 0.05) (Table 3).

**Table 3:** Bivariate analysis between pre-operative child's behaviour and potential factors over time (complete data).

Factors	0 M (*n* = 300)						18 M (*n* = 209)					
	Pre-operative VBRS			Post-operative VBRS			Pre-operative VBRS			Post-operative VBRS		
	Total cooperation (0)	Pre-cooperative (1–5)	*p*-value	Total cooperation (0)	Pre-cooperative (1–5)	*p*-value	Total cooperation (0)	Pre-cooperative (1–5)	*p*-value	Total cooperation (0)	Pre-cooperative (1–5)	*p*-value
	% (*n*)	% (*n*)		% (*n*)	% (*n*)		% (*n*)	% (*n*)		% (*n*)	% (*n*)	
**Gender**			*0*.*877*^a^			*0*.*034*^a^^,^*			*0*.*123*^b^			*0*.*450*^b^
Male	82.7 (134)	17.3 (28)		84.0 (136)	16.0 (26)		96.4 (106)	3.6 (4)		95.5 (105)	4.5 (5)	
Female	84.1 (116)	15.9 (22)		92.0 (127)	8.0 (11)		100.0 (99)	0.0 (0)		98.0 (97)	2.0 (2)	
**Place of birth** ^e^			*1*.*000*^b^			*1*.*000*^b^			*1*.*000*^b^			*1*.*000*^b^
Hong Kong	83.8 (238)	16.2 (46)		87.7 (240)	12.3 (35)		98.0 (194)	2.0 (4)		96.5 (191)	3.5 (7)	
Others	83.3 (10)	16.7 (2)		91.7 (11)	8.3 (1)		100.0 (8)	0.0 (0)		100.0 (8)	0.0 (0)	
**Brushing frequency** ^e^			*<0*.*001*^b^^,^*			*<0*.*001*^b^^,^*			*0*.*005*^b^^,^**			*0*.*558*^b^
Less than once a day	85.0 (17)	15.0 (3)		95.0 (19)	5.0 (1)		86.7 (13)	13.3 (2)		93.3 (14)	6.7 (1)	
Once a day	76.6 (72)	23.4 (22)		78.7 (74)	21.3 (20)		96.2 (50)	3.8 (2)		98.1 (51)	1.9 (1)	
Twice a day	88.0 (161)	12.0 (22)		92.9 (170)	7.1 (13)		100.0 (140)	0.0 (0)		96.4 (135)	3.6 (5)	
More than twice a day	0.0 (0)	100.0 (3)		0.0 (0)	100.0 (3)		100. 0 (2)	0.0 (0)		100.0 (2)	0.0 (0)	
**Supervised toothbrushing** ^e^			*0*.*338*^a^			*0*.*204*^b^			*0*.*499*^b^			*0*.*570*^b^
Never	89.5 (17)	10.5 (2)		94.7 (18)	5.3 (1)		100.0 (16)	0.0 (0)		93.8 (15)	6.3 (1)	
Sometimes	86.5 (90)	13.5 (14)		91.3 (95)	8.7 (9)		96.3 (77)	3.8 (2)		97.5 (78)	2.5 (2)	
Always	80.7 (142)	19.3 (34)		84.7 (149)	15.3 (37)		99.1 (112)	0.9 (1)		96.5 (109)	3.5 (4)	
**Toothpastes** ^e^			*0*.*149*^b^			*0*.*139*^b^			*0*.*101*^b^			*1*.*000*^b^
No toothpaste	86.4 (19)	13.6 (3)		86.4 (19)	13.6 (3)		100.0 (13)	0.0 (0)		100.0 (13)	0.0 (0)	
Child non fluoridated toothpaste	78.8 (89)	21.2 (24)		84.1 (95)	15.9 (18)		95.7 (66)	4.3 (3)		95.7 (66)	4.3 (3)	
Child fluoridated toothpaste	86.9 (119)	13.1 (18)		90.5 (124)	9.5 (13)		100.0 (108)	0.0 (0)		96.3 (104)	3.7 (4)	
Adult fluoridated toothpaste	50.0 (1)	50.0 (1)		50.0 (1)	50.0 (1)		100.0 (1)	0.0 (0)		100.0 (1)	0.0 (0)	
**Snacking frequency** ^e^			*0*.*087*^a^			*0*.*411*^b^			*0*.*737*^b^			*0*.*548*^b^
Less than once a day	79.2 (38)	20.8 (10)		87.5 (42)	12.5 (6)		100.0 (35)	0.0 (0)		100.0 (35)	0.0 (0)	
Once a day	78.4 (87)	21.6 (24)		83.8 (93)	16.2 (18)		98.6 (70)	1.4 (1)		94.4 (67)	5.6 (4)	
a day	90.6 (96)	9.4 (10)		90.6 (96)	9.4 (10)		97.3 (73)	2.7 (2)		97.3 (73)	2.7 (2)	
than twice a day	82.9 (29)	17.1 (6)		91.4 (32)	8.6 (3)		96.4 (27)	3.6 (1)		96.4 (27)	3.6 (1)	
**Night-bottle habit** ^e^			*0*.*882*^a^			*0*.*474*^b^			*0*.*025*^b^^,^*			*0*.*437*^b^
Never	82.6 (166)	17.4 (35)		86.4 (174)	13.4 (27)		99.3 (145)	0.7 (1)		95.9 (140)	4.1 (6)	
Yes	85.3 (29)	14.7 (5)		94.1 (32)	5.9 (2)		90.0 (18)	10.0 (2)		95.0 (19)	5.0 (1)	
Unwinded	84.6 (55)	15.4 (10)		87.7 (57)	12.3 (8)		97.7 (42)	2.3 (1)		100 (43)	0.0 (0)	
**Previous dental visit** ^e^			*0*.*804*^a^			*0*.*780*^b^			*1*.*000*^b^			*0*.*529*^b^
No	83.1 (222)	16.9 (45)		87.3 (233	12.7 (34)		97.9 (184)	2.1 (4)		96.8 (182)	3.2 (6)	
Yes	84.8 (28)	15.2 (5)		90.9 (30)	9.1 (3)		100.0 (21)	0.0 (0)		95.2 (20)	4.8 (1)	
**Father age** ^e^			*0*.*503*^b^			*0*.*559*^b^			*0*.*497*^b^			*0*.*756*^b^
< 30 years old	71.4 (10)	28.6 (4)		78.6 (11)	21.4 (3)		100.0 (7)	0.0 (0)		100.0 (7)	0.0 (0)	
30–39 years old	83.8 (124)	16.2 (24)		87.2 (129)	12.8 (19)		99.1 (106)	0.9 (1)		97.2 (104)	2.8 (3)	
40–49 years old	85.4 (88)	14.6 (15)		90.3 (93)	9.7 (10)		95.7 (67)	4.3 (3)		97.1 (6.8)	2.9 (2)	
> 50 years	89.3 (25)	10.7 (3)		89.3 (25)	10.7 (3)		100.0 (21)	0.0 (0)		95.2 (20)	4.8 (1)	
**Mother's age** ^e^			*0*.*077*^a^			*0*.*338*^a^			*0*.*498*^b^			*0*.*842*^b^
<30 years old	71.1 (27)	28.9 (11)		81.6 (31)	18.4 (7)		100.0 (20)	0.0 (0)		100.0 (20)	0.0 (0)	
30–39 years old	84.7 (166)	15.3 (30)		87.2 (171)	12.8 (25)		98.6 (139)	1.4 (2)		96.5 (136)	3.5 (5)	
40–49 years old	88.3 (53)	11.7 (7)		91.7 (55)	8.3 (5)		95.5 (42)	4.5 (2)		95.5 (42)	4.5 (2)	
>50 years	0.0 (0)	0.0 (0)		0.0 (0)	0.0 (0)		0.0 (0)	0.0 (0)		0.0 (0)	0.0 (0)	
**Household income (HK per month**)^e^			*0*.*176^†^*			*0*.*182*^b^			*0*.*842*^b^			*0*.*193*^b^
Below HK$20,001	75.4 (43)	24.6 (14)		80.7 (46)	19.3 (11)		97.1 (34)	2.9 (1)		94.3 (33)	5.7 (2)	
$20,000–30,000	86.6 (84)	13.4 (13)		88.7 (86)	11.3 (11)		98.4 (63)	1.6 (1)		100.0 (64)	0.0 (0)	
$30,001–40,000	90.2 (37)	9.8 (4)		95.1 (39_	4.9 (2)		100. 0 (32)	0.0 (0)		96.9 (31)	3.1 (1)	
$40,000 or above	81.3 (65)	18.8 (15)		85.0 (239)	13.1 (36)		96.7 (59)	3.3 (2)		95.1 (58)	4.9 (3)	
**Father's education level** ^e^			*0*.*092*^b^			*0*.*474*^b^			*0*.*133*^b^			*0*.*165*^b^
Primary	83.3 (5)	16.7 (1)		83.3 (5)	16.7 (1)		80.0 (4)	20.0 (1)		80.0 (4)	20.0 (1)	
Secondary	78.8 (119)	21.2 (32)		85.4 (129)	14.6 (22)		98.0 (97)	2.0 (2)		98.0 (97)	2.0 (2)	
Tertiary	87.3 (110)	12.7 (16)		88.0 (112)	11.1 (14)		98.9 (93)	1.1 (1)		95.7 (90)	4.3 (4)	
Postgraduate	100.0 (0)	0.0 (0)		100 (12)	11.1 (22)		100.0 (8)	0.0 (0)		100.0 (8)	0.0 (0)	
**Mother's education level** ^e^			*0*.*312*^b^			*0*.*044*^b,^*			*0*.*189*^b^			*0*.*205*^b^
Primary	63.3 (7)	36.4 (4)		63.6 (7)	36.4 (4)		83.3 (5)	16.7 (1)		83.3 (5)	16.7 (1)	
Secondary	84.3 (129)	15.7 (24)		89.5 (137)	10.5 (16)		98.1 (106)	1.9 (2)		96.3 (104)	3.7 (1)	
Tertiary	85.6 (95)	14.4 (16)		86.5 (96)	13.5 (15)		98.7 (78)	1.3 (1)		98.7 (78)	1.3 (1)	
Postgraduate	86.7 (13)	13.3 (2)		100.0 (15)	0.0 (0)		100.0 (10)	0.0 (0)		100.0 (10)	0.0 (0)	
**Number of siblings** ^e^			*0*.*710*^a^			*0*.*399*^b^			*0*.*125*^b^			*0*.*038*^b^^,^*
Only child	82.8 (77)	17.2 (16)		86.0 (80)	14.0 (13)		96.8 (60)	3.2 (2)		91.9 (57)	8.1 (5)	
2 children	85.2 (115)	14.8 (20)		91.1 (123)	8.9 (12)		100.0 (103)	0.0 (0)		99.0 (102)	1.0 (1)	
3 children or more	80.0 (32)	20.0 (8)		85.0 (34)	15.0 (6)		96.2 (25)	3.8 (1)		96.2 (25)	3.8 (1)	
	***Mean (SD)***	***Mean (SD)***		***Mean (SD)***	***Mean (SD)***		***Mean (SD)***	***Mean (SD)***		***Mean (SD)***	***Mean (SD)***	
**Age (months)** ^e^	49.8 (6.3)	48.4 (5.6)	*0*.*148*^c^	49.7 (6.3)	49.1 (5.5)	*0*.*565*^c^	50.8 (5.9)	47.3 (4.6)	*0*.*231*^c^	50.7 (5.9)	52.2 (5.3)	*0*.*486*^c^
**dft**	1.5 (3.2)	0.8 (1.7)	*0*.*267*^d^	1.5 (3.2)	0.7 (1.5)	*0*.*164*^d^	2.2 (3.5)	3.5 (4.4)	*0*.*590*^d^	2.2 (3.5)	4.0 (3.4)	*0*.*102*^d^
**dt**	1.5 (3.2)	0.8 (1.7)	*0*.*271*^d^	1.5 (3.1)	0.7 (1.5)	*0*.*168*^d^	1.8 (3.1)	3.5 (4.4)	*0*.*471*^d^	*1.9* (*3.1)*	*3.0* (*3.3)*	*0*.*134*^d^
**ft**	0.0 (0.1)	0.0 (0.0)	*0*.*437*^d^	0.0 (0.1)	0.0 (0.0)	*0*.*515*^d^	0.4 (1.6)	0.0 (0.0)	*0*.*469*^d^	0.3 (1.5)	1.0 (0.4)	*0*.*124*^d^
**VPI** ^e^	35.2 (16.5)	35.2 (20.0)	*0*.*989*^c^	36.9 (16.5)	31.0 (17.8)	*0*.*506*^d^	38.0 (14.8)	53.8 (30.1)	*0*.*372*^c^	38.1 (14.8)	43.3 (27.1)	*0*.*629*^c^

SD, standard deviation; VBRS, venham behavioural rating scale.

a *p*-values derived from Chi-square statistics.

b *p*-values derived from Fisher-exact statistics.

c *p*-value derived from independent *T*-test.

d *p*-value derived from Mann–Whitney *U*-test.

e undisclosed data were removed from the analyses.

* *p* < 0.05.

** *p* < 0.01.

*** *p* < 0.001.

At 18 months, only 4 out of 209 children exhibited uncooperative behaviour before the dental procedure, while 7 displayed uncooperative behaviour post-operatively. Following multiple imputation, statistical significance was observed across all 20 imputations (100%) regarding the mean dft and dt levels (*p* < 0.05). Children with higher mean dft and dt scores demonstrated increased uncooperative behaviour both before and after the dental examination. In 85% (17/20) of the imputations, children displaying total cooperation had significantly higher baseline ft scores pre-operatively (*p* < 0.05), although this association was not observed post-operatively. Toothbrushing frequency and night bottle feeding habits were found to have significant associations with uncooperative behaviours at 18 months in the complete dataset (Table 3). However, these factors, along with other sociodemographic factors, oral health habits, reported DFA, age and VPI scores, no longer showed significant associations with both pre-operative and post-operative behaviours in the pooled dataset after multiple imputation (*p* > 0.05).

## 4 Discussion

Families in Hong Kong appear to place limited importance on dental visit for children by age one, as evidenced by the study's findings showing over 89% of preschool children in the cohort had never visited a dentist at the initial assessment. In contrast, there is a strong cultural emphasis on early education, with nearly 100% enrolment in kindergartens (23), despite it not being mandatory. Although most children in the study experienced their first dental visit at baseline, they exhibited minimal fearful behaviours, with only 15.7% and 16.7% displaying uncooperative behaviours during the initial dental examination, as assessed by the Frankl and Venham scales. The prevalence of DFA found in this study was much lower than the reported DFA of 35% in dental settings and 23% in non-dental settings (3). This could be attributed to the outreach being conducted in a familiar kindergarten setting on a typical school day, with the presence of teachers and classmates providing a sense of comfort and familiarity, potentially enhancing their cooperation during the examination (6). At the same time, the implementation of various non-pharmacological behaviour management interventions, such as audio-visual distraction and positive reinforcement, has been effective in reducing dental anxiety in young children (24). The utilisation of audio-visual aids during dental examinations in the outreach setting played a crucial role in alleviating children's dental anxiety by redirecting their focus away from dental procedures, leading to a significant improvement in cooperation among preschool children (25).

The current study utilised three methods to assess on-site dental fear and anxiety (DFA) and pain levels: two behavioral scales administered by an operator and one self-reported pain scale. This comprehensive assessment approach offers a more holistic understanding of children's dental experiences and supports the development of improved treatment strategies. The FBRS and VBRS have a proven track record of effectively measuring children's direct behavioral responses during dental procedures. However, these results should be interpreted with caution, as behavioral ratings do not constitute a direct measure of dental fear and anxiety levels. Nonetheless, the use of the FBRS and VBRS remains one of the most practical and feasible approaches for assessing DFA in young children, who frequently lack the verbal and expressive capacity to communicate their dental fear and anxiety as adults typically do. The WBFS allows children to communicate their pain intensity through facial expressions. However, the WBFS depends on children's ability to identify with specific images, which may not always be accurate and can be influenced by a “middle bias”—particularly in very young children who tend to select extreme faces. As children develop cognitively, their ability to choose faces closer to the middle of the scale improves. Additionally, increased familiarity with dental check-ups over time may contribute to the conflicting results observed over the 18-month period.

There was a notable improvement in child cooperation as they matured, evidenced by significant changes in FBRS and VBRS behaviours over the 18 months period. These scales reflected significant pre-operative and post-operative differences when evaluated at different time points. A higher proportion of children demonstrated greater cooperation at 6 and 12 months compared to the baseline, similar to the findings reported by Chan et al. and Raj et al. (26, 27). Older preschool children often exhibit less dental fear and anxiety due to more mature cognitive functioning (28, 29). The reduction in anxiety may also be attributed to cognitive development of the children over the long 18-month review period. Additionally, in this study where the same children were reviewed every 6 months, repeated exposure to dental visits may have also played a role in enhancing their cooperativeness. This improvement suggests that prior experiences help children recognize the non-threatening nature of dental visits and better cope with stressful procedures. Overall, these findings support the notion that regular dental outreach programs are beneficial in fostering and reinforcing positive dental experiences for preschool children. However, these findings should be considered carefully due to the potential for selection bias, as indicated by previous statistical analyses revealing significant differences in confounding factors between children who were lost to follow-up and those who remained. The observed improvements in cooperation over time may partly result from the selective retention of more cooperative children, rather than representing genuine longitudinal changes across the entire cohort.

Similar to previous studies conducted in similar age groups, children with higher dft scores were observed to exhibit more DFA (30, 31). One possible explanation is that they may be aware of existing oral issues in their mouth, leading to feelings of anxiety (24). Alternatively, their visits to the clinic for treatments could contribute to the development of DFA during dental appointments (30, 31). Conversely, children with higher ft scores showed lower levels of DFA (30). The presence of intact fillings indicated that these children could manage the restorative procedures well, suggesting a lower DFA level.

Factors such as brushing only once a day and the presence of night bottle feeding habits were found to be significantly correlated with uncooperative behaviours. These habits may be linked to parenting style and children's individual characteristics, as strong-willed children who demonstrated uncooperative dental behaviours (32, 33) may resist routine toothbrushing and give up the night bottle. Additionally, being the only child was identified as a significant factor in this study, consistent with findings from another study involving children aged between 5 and 7 (34). However, these results should be interpreted cautiously due to the limited number of children displaying uncooperative behaviour in the study, and further validation is necessary with a larger sample size. Both CDAS and CFSS-D scores are self-reported assessments of participants' own DFA. The advantages include minimising outcome assessment bias. However, potential drawbacks may include recall bias and susceptibility to cognitive function and comprehension limitations, particularly in young children. Aside from recall bias, self-reported DFA measures may also have limited accuracy in young children, given the cognitive and developmental limitations associated with self-reporting in preschool-aged children. Hence CFSS-D was only one of the methods used to assess baseline DFA of child participants in this research alongside clinical evaluations. The associations between change in DFA level with the parental CDAS and CFSS-DS should also be explored with a larger sample size for more robust findings. Further research should target on parenting styles and other child-related factors, and should be conducted with a larger sample size.

Another limitation that compromises the robustness of the findings is the dropout rate of 30.3%, which exhibited significant differences in sociodemographic factors and baseline cooperativeness between those who remained in the study and those who were lost to follow up. Parents with lower dental awareness were more likely to have their children absent on the day of the dental examination and less likely to reinforce toothbrushing twice per day or use fluoridated toothpaste. Studies have also shown that parental dental awareness is strongly correlated with the children's DFA (35, 36). In addition to absences on the examination day, the unexpected dropout rate may be attributed to students withdrawing from their participating kindergartens. According to Hong Kong government statistics from 2022, there was a net outflow of 60,000 Hong Kong residents, with approximately 6% (at least 6,500) of kindergarten children deciding not to continue their studies in Hong Kong (37, 38). Multiple imputation is a commonly used statistical method for handling missing data in clinical trials and was adopted in the current study to handle the missing data due to lost to follow-up (39). It can help reduce bias, account for uncertainty, and preserve sample size (20). This method is achievable with recently developed software programs. Nevertheless, it would be beneficial to corroborate the present results with a more extensive sample size to accommodate the dropout variation. Due to the low prevalence of uncooperative behaviours, bivariate analyses were utilised in the regression models. However, this would potentially limit the ability to adjust for potential confounders, and introduce bias into the evaluation. Changes in the children's sociodemographic background, oral health factors, and other confounding variables may also vary over the extended review period, representing another potential limitation of the study; particularly since baseline information was not further verified at other timepoints for all participants in the cohort. Potential recall bias from self-reported information on DFA and baseline data collected through parental-administered questionnaires may also be a limitation. Since the research was conducted in kindergarten settings, there might be limited generalizability of the findings when applied to conventional clinical dental environments.

## 5 Conclusion

This study highlights the decrease in the prevalence of dental fear of preschool children towards dental examination in a familiar kindergarten and nursery setting over time. The findings suggest a lower prevalence of dental fear compared to global averages, emphasising the benefit of non-clinical settings for initial dental encounters Significant associations were found between past dental experience and untreated dental caries with DFA among preschool children. However, additional research is necessary to identify risk factors associated with dental anxiety and fear among preschool children more comprehensively, utilising a larger sample size.

## Data Availability

The anonymized raw data supporting the conclusions of this article are available from the corresponding author upon reasonable request.
